# Schwann Cells in the Aganglionic Colon of Hirschsprung Disease Can Generate Neurons for Regenerative Therapy

**DOI:** 10.1093/stcltm/szac076

**Published:** 2022-11-02

**Authors:** Weikang Pan, Ahmed A Rahman, Rhian Stavely, Sukhada Bhave, Richard Guyer, Meredith Omer, Nicole Picard, Allan M Goldstein, Ryo Hotta

**Affiliations:** Department of Pediatric Surgery, Massachusetts General Hospital, Harvard Medical School, Boston, MA, USA; Department of Pediatric Surgery, The Second Affiliated Hospital of Xi’an Jiaotong University, Shaanxi, People’s Republic of China; Department of Pediatric Surgery, Massachusetts General Hospital, Harvard Medical School, Boston, MA, USA; Department of Pediatric Surgery, Massachusetts General Hospital, Harvard Medical School, Boston, MA, USA; Department of Pediatric Surgery, Massachusetts General Hospital, Harvard Medical School, Boston, MA, USA; Department of Pediatric Surgery, Massachusetts General Hospital, Harvard Medical School, Boston, MA, USA; Department of Pediatric Surgery, Massachusetts General Hospital, Harvard Medical School, Boston, MA, USA; Department of Pediatric Surgery, Massachusetts General Hospital, Harvard Medical School, Boston, MA, USA; Department of Pediatric Surgery, Massachusetts General Hospital, Harvard Medical School, Boston, MA, USA; Department of Pediatric Surgery, Massachusetts General Hospital, Harvard Medical School, Boston, MA, USA

**Keywords:** enteric nervous system, Hirschsprung disease, Schwann cells, cell therapy

## Abstract

Cell therapy offers the potential to replace the missing enteric nervous system (ENS) in patients with Hirschsprung disease (HSCR) and to restore gut function. The Schwann cell (SC) lineage has been shown to generate enteric neurons pre- and post-natally. Here, we aimed to isolate SCs from the aganglionic segment of HSCR and to determine their potential to restore motility in the aganglionic colon. Proteolipid protein 1 (PLP1) expressing SCs were isolated from the extrinsic nerve fibers present in the aganglionic segment of postnatal mice and patients with HSCR. Following 7-10 days of in vitro expansion, HSCR-derived SCs were transplanted into the aganglionic mouse colon ex vivo and in vivo. Successful engraftment and neuronal differentiation were confirmed immunohistochemically and calcium activity of transplanted cells was demonstrated by live cell imaging. Organ bath studies revealed the restoration of motor function in the recipient aganglionic smooth muscle. These results show that SCs isolated from the aganglionic segment of HSCR mouse can generate functional neurons within the aganglionic gut environment and restore the neuromuscular activity of recipient mouse colon. We conclude that HSCR-derived SCs represent a potential autologous source of neural progenitor cells for regenerative therapy in HSCR.

## Graphical Abstract

**Graphical Abstract:**
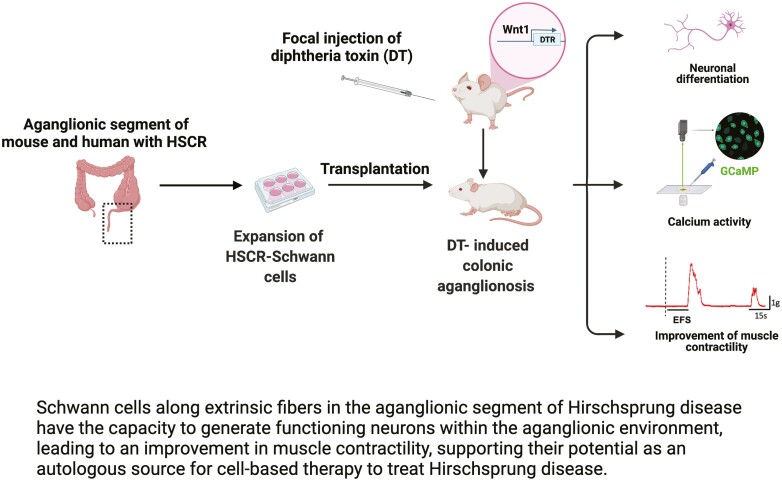

**Significance Statement**

Hirschsprung disease (HSCR) is a congenital disorder characterized by failure of the enteric nervous system to develop in the distal part of the intestine. Surgical removal of the aganglionic segment is the only treatment currently for HSCR, but outcomes are often unsatisfactory. Cell therapy offers the potential to restore colorectal function. We show that Schwann cells residing along extrinsic nerves in the aganglionic bowel can be isolated and expanded in culture. These cells can be transplanted into the aganglionic colon of mice with HSCR, where they exhibit neuronal activity and restore colonic contractility, offering a potential new autologous source of cells for regenerative therapy.

## Introduction

Innervation of the gastrointestinal (GI) tract is provided by two distinct neuronal populations, whose cell bodies lie either within the gut wall (intrinsic; enteric nervous system (ENS)) or outside the GI tract (extrinsic; sympathetic and parasympathetic neurons). The ENS regulates multiple critical functions of the GI tract largely independent of the control of the central nervous system.^1^ Therefore, abnormalities in the ENS can cause serious morbidity, including Hirschsprung disease (HSCR). HSCR is a congenital disease affecting 1 in 5000 children and characterized by the absence of ENS along variable lengths of the distal bowel due to failure of neural crest-derived precursors to complete their colonization of the developing intestine.^2^ The aganglionic intestine is functionally obstructed, and treatment involves surgical resection of this portion of bowel. While surgery is lifesaving, many children have persistent GI problems, including constipation, fecal incontinence, and enterocolitis.^3^

Cell therapy offers the potential to replace missing neurons in the intestine and ameliorate the functional deficits. Enteric neural stem/progenitor cells can be isolated from the postnatal rodent^4-7^ and human intestine,^8-11^ even from mucosal biopsy samples^12^ or from aganglionic colon resected from patients with HSCR.^13^ The latter observation led us to hypothesize that autologously-derived neural progenitor cells could be obtained from the bowel resected during HSCR surgery. Hence, post–pullthrough neurointestinal disorders could be treated by transplanting these autologous neural progenitors, which would avoid the immunogenic concerns associated with other cell sources.^14^

There has been growing evidence that the Schwann cell lineage has the potential to contribute to developing enteric neurons pre- and postnatally.^15,16^ Aganglionic bowel in HSCR lacks intrinsic ENS, whereas extrinsic-derived neural fibers are present and often hypertrophic.^17,18^ It has been reported that Schwann cells (SCs) reside within these extrinsic nerves and possess neurogenic potential^15,19,20^ and this has been confirmed in the fibers projecting to the aganglionic bowel.^16,21^

We utilized an animal model of HSCR in which proteolipid protein 1 (PLP1) expressing SCs are fluorescently labeled for isolation and expansion of HSCR-derived Schwann cells (HSCR-SCs) in culture. These HSCR-SCs were transplanted to an experimental model of colonic aganglionosis, where they demonstrate the ability to engraft and restore contractile function in the aganglionic smooth muscle. Our results illustrate that extrinsic nerve-derived neuronal precursors are present in the aganglionic bowel and have the potential to serve as an autologous source of neurons to restore innervation in the aganglionic bowel.

## Materials and Methods

### Animals

All animal protocols were approved by the Institutional Animal Care and Use Committee at Massachusetts General Hospital (Protocols #2009N000239 and #2013N000115). The following mouse lines were obtained from Jackson Laboratory (Bar Harbor, ME, USA): *Tau*^*GFP/GFP*^ mice, *Ednrb*^*+/−*^ mice, *Wnt1::Cre* mice, *R26-iDTR* mice, and *PC::G5-tdT* mice (Supplementary Table S1). *Plp1-GFP* mice were kindly gifted by Dr Wendy Macklin, University of Colorado.^22^ The various breeding schemes and genotypes of controls are summarized in Table 1. *Wnt1::Cre* mice were crossed with *R26-iDTR* mice to obtain *Wnt1-Cre;R26-iDTR* (Cre^+^) mice (hereafter referred to as Wnt1-iDTR). *R26-iDTR* mice (Cre^-^) were used as control.

**Table 1.** Parental crosses and offspring used for the experiments.

Parent cross	Genotype of the offspring	Descriptions in the manuscript	Figures
*Tau* ^*GFP/GFP*^ *;Ednrb* ^*+/−*^ X *Tau*^*GFP/GFP*^*;Ednrb*^*+/−*^	*Tau* ^*GFP/GFP*^ *;Ednrb* ^*−/−*^	Tau^GFP^;Ednrb^−/−^ or Tau^GFP^ HSCR	1
	*Tau* ^*GFP/GFP*^ *;Ednrb* ^*+/+*^	Tau^GFP^;Ednrb^+/+^ or Tau^GFP^ wild type	
*Plp1−GFP;Ednrb* ^*+/−*^ X *Plp1−GFP;Ednrb*^*+/−*^	*Plp1−GFP;Ednrb* ^*−/−*^	Plp1−GFP;Ednrb^−/−^ or Plp1−GFP HSCR	3, 5
	*Plp1−GFP;Ednrb* ^*+/+*^	Plp1−GFP;Ednrb^+/+^ or Plp1−GFP wild type	
*Wnt1::Cre;Ednrb* ^*+/−*^ X *PC::G5−tdT*^*+/−*^*;Ednrb*^*+/−*^	*Wnt1−PC::G5−tdT;Ednrb* ^*−/−*^	Wnt1−G5−tdT;Ednrb^−/−^ or Wnt1−G5−tdT HSCR	4, 5
	*Wnt1−PC::G5−tdT;Ednrb* ^*+/+*^	Wnt1−G5−tdT;Ednrb^+/+^ or Wnt1−G5−tdT wild type	
*Wnt1::Cre* X *R26−iDTR*	*Wnt1::Cre;R26−iDTR*	Wnt1−iDTR	5,6,7
	*R26−iDTR*	Cre-control	

### Tissue Dissection and Cell Culture

#### Mouse Tissues

Colonic tissues were dissected following euthanasia. Aganglionic segment was determined by the absence of ganglion cells. Longitudinal muscle-myenteric plexus (LMMP) layers containing hypertrophic nerve bundles were obtained. Tissues were minced with micro scissors and dissociated briefly with Dispase (250 μg/mL; STEMCELL Technologies, Vancouver, BC) and collagenase XI (1 mg/mL; Sigma–Aldrich, St. Louis, MO) at 37 °C for 10-15 min. A 40 μm cell strainer (Corning Inc, Corning, NY) was used to collect nerve bundles. Residual nerve fibers were digested with the same enzyme as above for an additional 10-15 min to dissociate into single-cell suspension.

Cells from the aganglionic segment of Tau^GFP^ Ednrb^−/−^ or Plp1-GFP Ednrb^−/−^ mice were sorted for GFP using a MoFloXDP cell sorter (Beckman Coulter). The GFP-positive cells were selected using a 530/30 filter set. Gating parameters were set using cells from wild-type gut and applied to increase the specificity of the selection of GFP positive and GFP negative cells.

GFP+ or tdTomato+ cells were plated in a flat bottom ultra-low attachment multiple well culture plate (Corning, NY) in cell culture media containing NeuroCult^TM^ Basal medium (STEMCELL Technologies, CA) supplemented with 20 ng/mL epidermal growth factor (STEMCELL Technologies), 10 ng/mL basic fibroblast growth factor (STEMCELL Technologies), 50 μL Heparin (STEMCELL Technologies), and 100 U/mL penicillin-streptomycin (Life Technologies).

#### Human Tissues

After obtaining human research approval (IRB protocol #2010P00669), the aganglionic colon was cut into ~1 cm^2^ pieces and washed 3 times in sterile Hanks’ Balanced Salt Solution (HBSS, Thermo Fisher, Waltham, MA). For enzymatic digestion, pieces of LMMP were digested in Liberase^TM^ Thermolysin High formulation (25 µg/mL, Roche, Indianapolis, IN) and Dispase (0.05 U/mL; STEMCELL Technologies) for 2-3.5 h in a humidified incubator at 37 °C. The dissociated tissues were filtered through a 70 µm cell strainer, and the counter-filtered material was collected and washed with sterile PBS. Under a light dissection microscope, nerve fiber bundles were manually collected based on morphology.

Nerve fiber bundles were cut into pieces and digested with the same enzyme above for an additional 10-15 min. These cells were placed into ultra-low attachment culture plates (6-well plate, Costar, ME) containing 1 mL of human neural proliferation medium supplemented with basic fibroblast growth factor (20 ng/mL, STEMCELL Technologies), epidermal growth factor (20 ng/mL, STEMCELL Technologies), heparin (0.0002%, STEMCELL Technologies), GlutaMAX (1%, Life Technologies), B27 supplement (1%, Thermo Fisher), Primocin, (1%, Invivogen, San Diego, CA), Metronidazole (50 µg/mL, Sigma–Aldrich) and FBS (5%, Thermo Fisher) in Dulbecco’s Modified Eagle Medium: Nutrient Mixture F-12 (DMEM/F12, Gibco, Life Technologies).

### Cell Transplantation to Aganglionic Mouse Colon Ex Vivo

NLBs were co-cultured with the isolated muscularis propria of aganglionic colon from *Ednrb*^−/−^ mice as previously described.^4^ LMMP of aganglionic colon were prepared as described above and placed on a filter paper with a rectangular window. A small pocket was created in the muscularis propria using fine forceps and NLBs were transplanted into the pocket, then cultured for 7 days in tissue culture media, consisting of 10% FBS and 1% penicillin/streptomycin in Dulbecco’s Modified Eagle Medium (DMEM).

### Cell Transplantation to DT-Induced Aganglionic Colon of Wnt1-iDTR Mice In Vivo

Focal colonic aganglionosis was created as described previously.^23^ Briefly, 3-month-old Wnt1-iDTR mice were microinjected with 4 μL of 0.5 μg/mL diphtheria toxin (DT) with India ink via laparotomy to the wall of mid-colon. One week after DT injection, up to 15 μL suspension containing ~80 NLBs was microinjected using NanoFil^TM^ micro syringe (33G, NF33BV-2, World Precision Instruments, Fl, USA) to the aganglionic colon identified by India ink.

### Tissue Preparation and Immunohistochemistry

Tissue preparation and immunohistochemistry were performed as previously described.^24^ Cells, LMMP, and full-thickness gut samples were fixed in 4% paraformaldehyde. For cryosections, full-thickness gut samples were embedded in 15% sucrose at 4 °C overnight, and then in 15% sucrose plus 7.5% gelatin at 37 °C for 1 h. Then the tissue was rapidly frozen at −50 °C in Nitrogen. Frozen sections were collected on glass slides at 12-14 μm thickness with a Leica CM3050 S cryostat (Leica, Buffalo Grove, IL). For immunohistochemistry, the samples were permeabilized with 0.1% Triton X-100 and blocked with 10% donkey serum for 30 min. Primary antibodies were diluted in 2% donkey serum, 0.01% Triton X-100 and included rabbit anti-p75 neurotrophin receptor (P75; 1:400; Promega, Madison, WI), mouse anti-neuronal class III β-tubulin (Tuj1; 1:400; conjugated to Alexa Fluor 546, Invitrogen), human anti-Hu (Anna1, 1:16 000, kindly gifted by Lennon lab), rabbit anti-glial fibrillary acidic protein (GFAP, 1:200, Agilent/Dako, Denmark), goat anti-GFP (1:400, Rockland, Limerick, PA), rabbit anti-glucose transporter type 1 (GLUT1, 1:200, Millipore), mouse anti-PLP1 (1:100, Thermo Fisher), rabbit anti-calretinin (1:200, Invitrogen), rabbit anti-smooth muscle actin (SMA; 1:200; Abcam, Cambridge, MA), and human cell nuclei antibody (HUNUC, 1:100, conjugated to Alexa Fluor 488, Millipore), and rabbit anti-synapsin 1 (1:800, Cell Signaling). Secondary antibodies used in this study are donkey anti-rabbit IgG (1:500; Alexa Fluor 488 and 546; Fisher Scientific Life Technologies), donkey anti-goat IgG (1:500; Alexa Fluor 488; Fisher Scientific Life Technologies), goat anti-mouse IgG (1:500; Alexa Fluor 488; Fisher Scientific Life Technologies), and donkey anti-human IgG (1:200, Alexa Fluor 488 and 647; Fisher Scientific Life Technologies). Cell nuclei were identified with DAPI solution (Vector Labs, Burlingame, CA) and mounted with Aquapoly/mount (Fisher Scientific Polysciences Inc.). Images were taken using a Nikon A1R laser scanning confocal microscope (Nikon Instruments, Melville, NY) or a Keyence BZX-700 All-In-One Microscopy (Keyence America Itasca, IL).

### Live Cell Imaging In Vitro and Ex Vivo

For imaging of in vitro cultured GCaMP5 cells, NLBs grown from Wnt1-G5-tdT HSCR mice were transferred to fibronectin-coated (1:500 of sterile PBS for 1h at 37 °C) cell culture plates and grown in BrainPhys^TM^ neuronal medium (STEMCELL Technologies) supplemented with N2 (1%, STEMCELL Technologies) and SM1 (2%, STEMCELL Technologies), for 7 days. The media was changed with Krebs’ solution (in mM: 118 NaCl, 4.7 KCl, 1.2 KH2PO4, 1.5 MgSO_4_, 25 NaHCO_3_, 11 D-Glucose, 2.5 and 2.5 CaCl_2_, pH 7.4) on the day of analysis. The L-type Ca^2+^ channel blocker nicardipine (2 µM, Sigma Aldrich) was added to limit the muscle contractions and to improve the stability for the analysis of calcium imaging data. The fluorescence of the GCaMP5 calcium indicator was recorded as a movie for 10 min at a 40-Hz sampling rate using the Keyence BZX-700 All-In-One Microscopy system. Different doses of ACh (Sigma–Aldrich) were added to the medium at 1 min (1 μM), 4 min (10 μM), and 7 min (25 μM) from the beginning of the recording.

For imaging of GCaMP5 cells transplanted into aganglionic colon ex vivo or in vivo, recipient’s colons were dissected and pinned on a sylgard-coated glass bottom dish (Dow Corning, Midland, MI, USA) superfused with Krebs’ solution. Live cell imaging was performed as above while stimulating with different EFS pulses (single: 30 V and 50 V; continuous: 50 V 300 µs pulse width at 5 Hz for 5 s) delivered by 2 parallel platinum electrodes placed on either side of colonic preparations. EFS was applied by a CS4+ constant voltage stimulator and MyoPulse software (DMT, Hinnerup, Denmark).

Levels of GFP intensity were quantified using ImageJ software (National Institutes of Health, Bethesda, MD, USA). Changes in the intensity of selected cells (by region of interest) were calculated and documented as relative fluorescence (ΔF/F0).

### Measurement of Muscular Contraction of the Colon

Experiments were performed using standard organ bath technique as described previously.^25^ Freshly excised distal colon was quickly placed in a Petri dish containing physiological Krebs’ solution. The colonic segment marked by Indian ink was cut into a 5-mm ring. The colonic rings were then mounted between 2 small metal hooks attached to force displacement transducers in a muscle strip myograph bath (Model 820 MS; Danish Myo Technology, Aarhus, Denmark) containing 7 mL of physiological Krebs’ solution (oxygenated with 95% O_2_ and 5% CO_2_) maintained at 37 °C. Then, the rings were stretched to give a basal tension of 0.5 g and were equilibrated for 60 min in Krebs’ solution changed at every 20 min. Force contraction of the circular smooth muscle was recorded and analyzed by using a Power Lab 16/35 data acquisition system (ADInstruments, NSW, Australia) and a computer via Lab Chart Pro Software v8.1.16 (ADInstruments, NSW, Australia). Tissue viability and integrity were checked by eliciting contraction response to 60 mM KCl. Colon segments were stimulated with pulse trains of 10-50 V for 30 s, with the pulse duration of 300 µs, at a frequency of 5 Hz by using a CS4+ constant voltage stimulator with Myo Pulse software (Danish Myo Technology, Aarhus, Denmark).

### Statistical Analysis

Data analyses were performed using Prism 9 (GraphPad Software, Inc., La Jolla, CA, USA) and presented as mean ± SD. NLBs size, numbers, and relative expression were compared using Student’s *t-*test. A one-way analysis of variance (ANOVA) was performed for multiple comparisons. For all analyses, *P* values <.05 were regarded as significant.

## Results

### Non-Neuronal Cells Along Extrinsic Nerves in the Aganglionic Colon of HSCR Mice can be Expanded in Culture and Give Rise to Neurons

**Figure 1.**
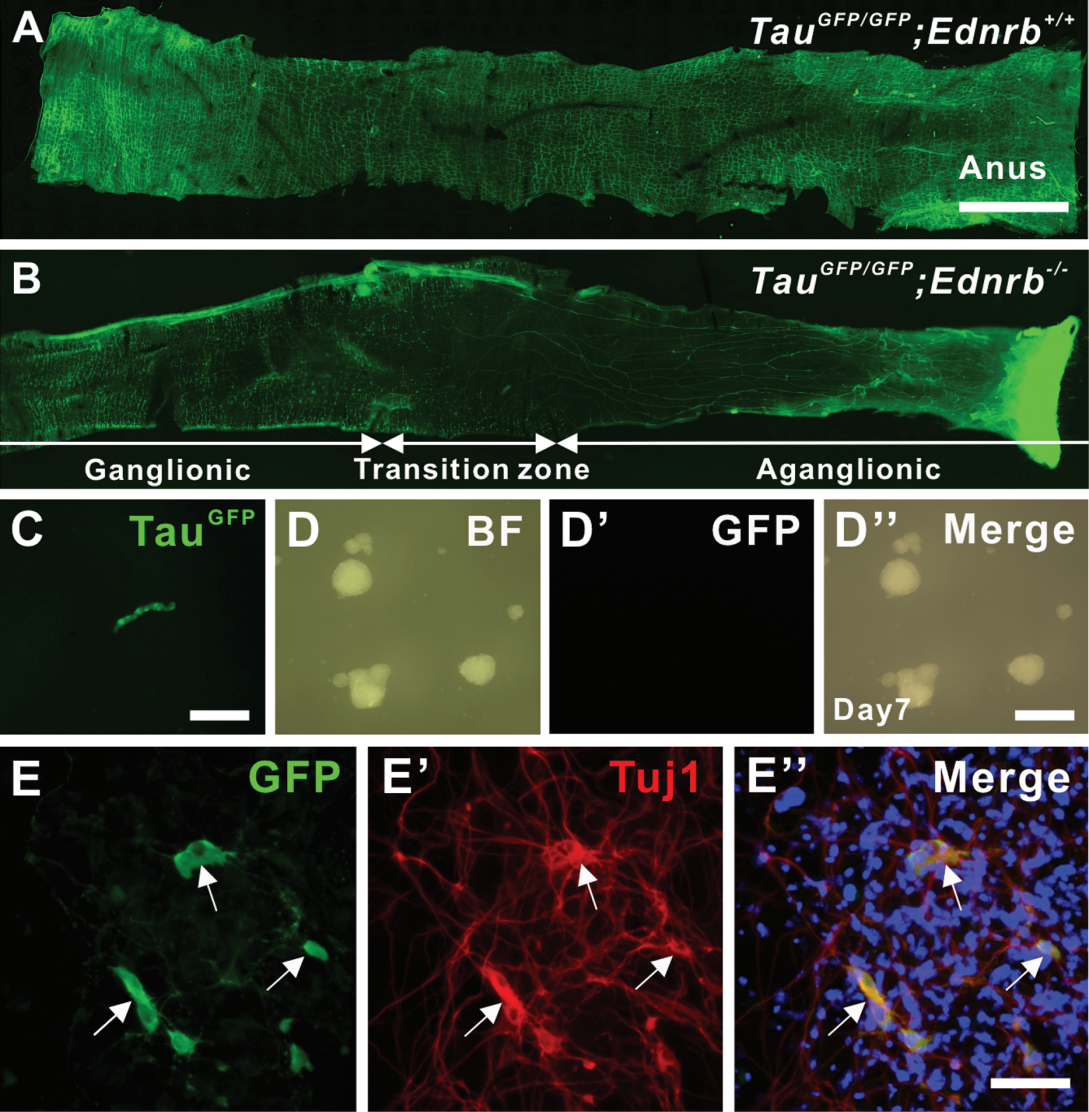
Non-neuronal cells in the extrinsic nerves in the aganglionic colon of HSCR mouse can be expanded in culture and give rise to neurons. Wholemount preparation of colon from *Tau*^*GFP/GFP*^*;Ednrb*^*+/+*^ (**A**) and *Tau*^*GFP/GFP*^*;Ednrb*^−*/*−^ (**B**) mouse. Hypertrophic nerve fibers (B, arrows) within the aganglionic segment are manually separated (**C**), dissociated and cultured to form NLBs (**D**-**D**ʹ). Those GFP negative NLBs contain neural progenitor cells that can give rise to neurons (**E**, GFP, arrows) with immunoreactivity for Tuj1 (**E**ʹ, arrows) following 7 days culture on fibronectin. Scale bars 1cm (A, B), 50 μm (D-Dʹʹ), 100 μm (C, E-Eʹʹ).

The aganglionic colon of patients with HSCR contains P75^+^ cells that possess characteristics of neural progenitors,^13^ but the origin and niche of those progenitors are not well investigated. We first addressed whether hypertrophic nerve fibers along the aganglionic colon of HSCR mouse contain neural progenitors using Tau^GFP^;Ednrb^−/−^ mice, a model of HSCR in which all neurons express green fluorescent protein (GFP). Enteric ganglia are seen along the entire colon of 2-week-old Tau^GFP^;Ednrb^+/+^ (Fig. 1A), whereas distal aganglionosis with GFP positive hypertrophic nerve bundles (Fig. 1B) are seen in Tau^GFP^;Ednrb^−/−^ mice. We mechanically dissected these hypertrophic fibers from 14-day-old Tau^GFP^;Ednrb^−/−^ mouse colon (Fig. 1C), followed by enzymatic dissociation and separation of the GFP positive and negative fractions using flow cytometry (1.4 ± 8.7 × 10^4^ GFP positive and 11.2 ± 5.2 × 10^4^ GFP negative cells were isolated, *n* = 3). Following the culture of both fractions, neurosphere-like bodies (NLBs) formed only from the GFP negative fraction (97.3 ± 18.7 spheroids from 5000 cells, with the average size of 44.6 ± 0.7 μm in diameter, Fig. 1D, 1D″). These GFP-negative NLBs were further cultured for 7 days on fibronectin-coated coverslips, and we observed that 23.5% of overall cells were GFP positive (Fig. 1E, arrows) with immunoreactivity for the pan-neuronal marker, Tuj1 (Fig. 1Eʹ, arrows). These results suggest that non-neuronal cells within the aganglionic region of HSCR mice can be expanded and possess neurogenic potential in culture.

### PLP1-GFP Schwann Cells Residing Along Colonic Extrinsic Nerve Fibers Exhibit Characteristics of Neural Progenitor Cells

**Figure 2.**
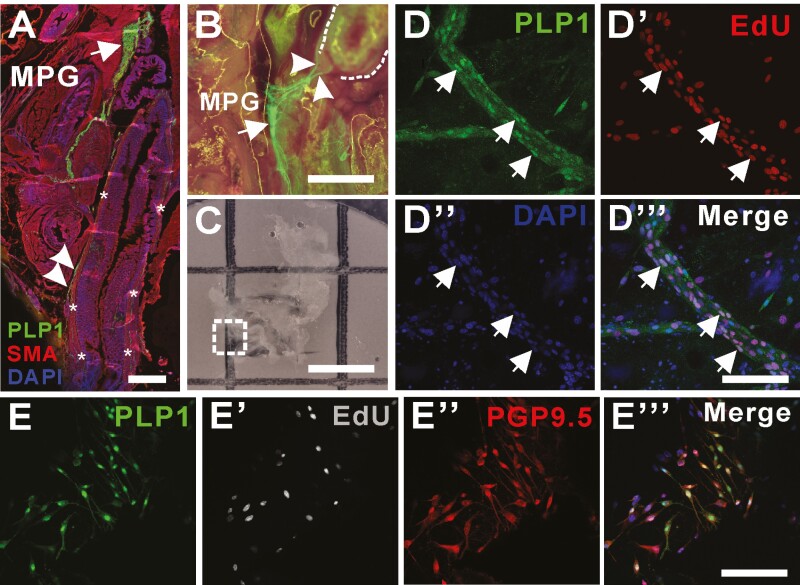
PLP1-GFP glial cells residing in the extrinsic nerve fibers have a capacity to proliferate in vivo. (**A**) Sagittal section of pelvic cavity of *Plp1-GFP* mouse including distal colon (asterisks) with immunostaining with SMA shows left major pelvic ganglia (MPG, **A** and **B**, arrows) contain PLP1 expressing glial cells and fibers (A, B, arrowheads) that extends to the distal colon (A, asterisks and B, dotted line). Dissection and staining (C, dotted box) of MPG from *Plp1-GFP* mouse in which EdU has been administered intraperitoneally demonstrate proliferative capacity of GFP positive cells in the extrinsic colonic fibers (D-Dʹʹʹ, arrows). MPG is dissected from *Plp1-GFP* mouse for ex vivo organ culture for up to 7 days allowing evaluation of their capacity to proliferate (Eʹ, EdU) and to differentiate into neurons (Eʹʹ, PGP9.5) in vitro. Scale bars 500 μm (**A**), 200 μm (**B**, **C**), 100 μm (**D**-**D**ʹʹʹ). 50 μm (**E**-**E**ʹʹʹ), EdU, 5-ethynyl-2ʹ-deoxyuridine. Abbreviations: MPG, major pelvic ganglia; SMA, smooth muscle actin.

Recent reports have demonstrated that glial cells and the Schwann cell (SC) lineage possess neurogenic potential in the postnatal intestine.^15,19,21^ We utilized *Plp1-GFP* mice, in which both enteric glial cells and SCs are labeled by GFP,^26,27^ to examine whether PLP1-expressing cells are able to proliferate and differentiate into neurons. The sagittal section of the pelvic region of 3-month-old Plp1-GFP wild-type mouse showed GFP expression in enteric glia within the colonic myenteric plexus (Fig. 2A, arrowheads) and SCs located in the major pelvic ganglia^22^ (Fig. 2A and B, arrows). Extramural fibers extending from the major pelvic ganglia to the distal colon (Fig. 2B, arrowheads) are also GFP positive, consistent with previous observations in which myelinating SCs in the extrinsic nerves express PLP1.^27^ 5-ethynyl-2ʹ-deoxyuridine (EdU) was administered daily to 1-month-old Plp1-GFP wild type mice for 5 days. Major pelvic ganglia were dissected and placed on filter paper (Fig. 2C) for further tissue processing and imaging. Extramural extrinsic fibers extending from the pelvic ganglia contain PLP1–positive SCs (Fig. 2C, dotted box magnified in 2D) that incorporate EdU (Fig. 2D, Dʹʹʹ, arrows), suggesting their proliferative potential in normal physiologic conditions *in vivo*.

To determine whether these PLP1 positive cells within the extrinsic fibers are neurogenic, the extrinsic fibers together with the pelvic ganglia of 1-month-old Plp1-GFP wild–type mice were carefully dissected and cultured for 7 days, with EdU added daily for the first 3 days. Immunohistochemical staining showed extensive migration of GFP–positive cells away from the fibers. Those extrinsic-derived SCs incorporated EdU and underwent neuronal differentiation, as shown by PGP9.5 expression (Fig. 2E, Eʹʹʹ), confirming that PLP1 expressing SCs within the extrinsic fibers are proliferative and neurogenic.

### PLP1 Positive Schwann Cells Residing Along Hypertrophic Nerve Fibers in HSCR Mice (HSCR-SCs) Can Proliferate and Differentiate into Neurons

**Figure 3.**
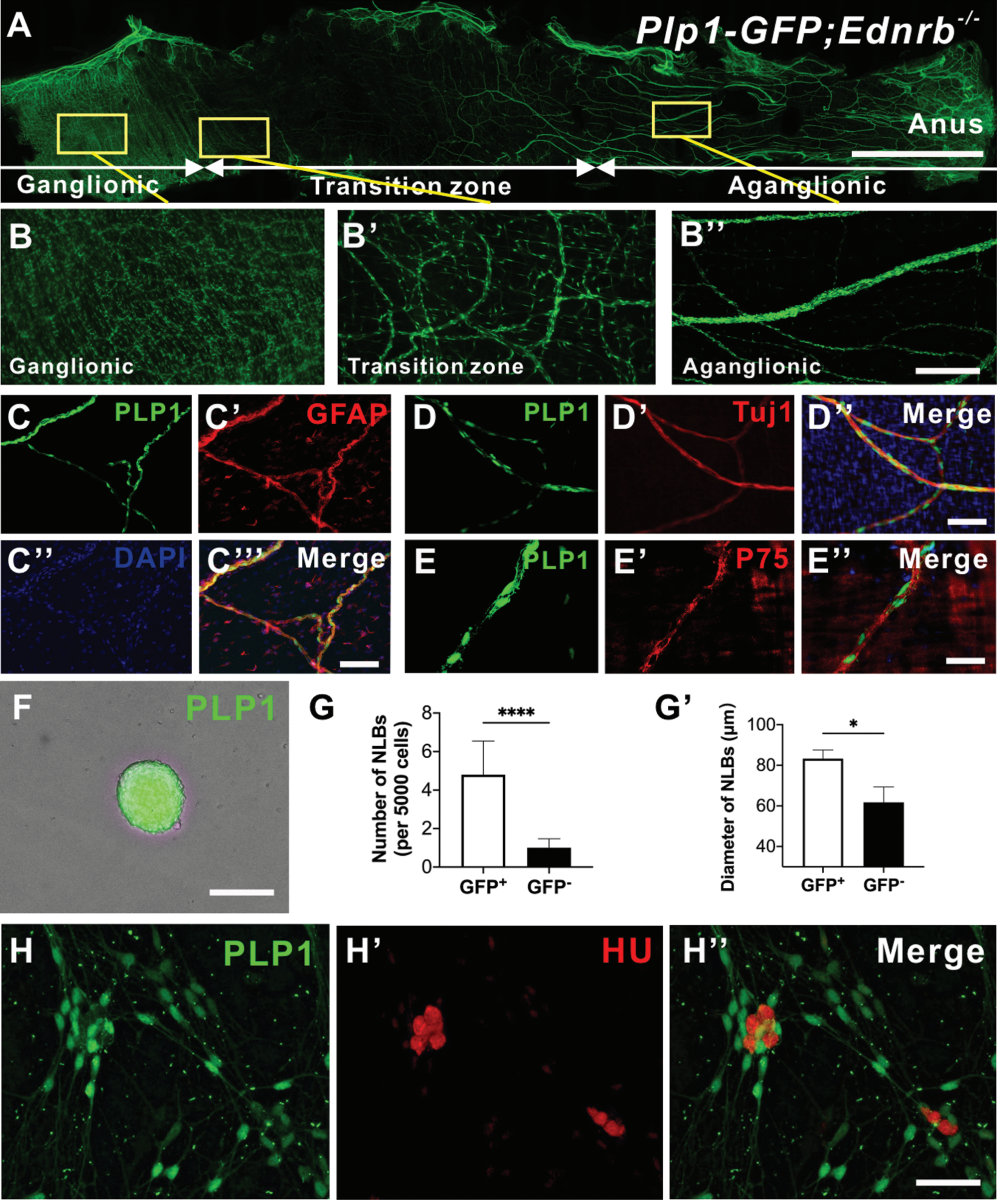
PLP1-GFP Schwann cells residing in hypertrophic nerve fibers in HSCR mice can proliferate and differentiate into neurons in vitro. Representative image of colon from *Plp1-GFP;Ednrb*^*−−-*^ mouse (**A**) with high power images of ganglionic (**B**), transition zone (**B**ʹ), and aganglionic segment (Bʹʹ’). Wholemount staining of extrinsic fibers in the agangionic colon of the *Plp1-GFP;Ednrb*^*−−-*^ mouse with GFAP (**C-C**ʹʹʹ), Tuj1 (**D-D**ʹʹ) and P75 (**E**-**E**ʹʹ). Hypertrophic fibers in the aganglionic segment of *Plp1-GFP;Ednrb*^*−/−*^ mice were mechanically separated, sorted and cultured to form GFP positive NLBs (**F**). GFP positive cells generated significantly more (**G**, *n* = 5, ****P < .0001) and larger NLBs (**G**ʹ, *n* = 5, * *P* < .05) compared to GFP negative fraction. PLP1-GFP cells are able to generate neurons in vitro confirmed by immunoreactivity for Hu (**H-H**ʹʹ). Data are represented as mean ± standard deviation from 5 mice per group by Student’s *t* test. Scale bars 1 cm (A), 50 μm (B), 100 μm (C-F, H-Hʹʹ).

Given the observations that the non-neuronal lineage within the hypertrophic bundles in the aganglionic segment of HSCR can generate neurons (Fig. 1), and that SCs in the extrinsic colonic fibers exhibit characteristics of neural progenitor cells (Fig. 2), we hypothesized that SCs in the hypertrophic bundles in HSCR represent neural progenitors. To test this, we generated a mouse model of HSCR in which SCs express GFP by crossing *Plp1-GFP* mice and *Ednrb*^+/-−^ mouse line. Fourteen days old *Plp1-GFP;Ednrb*^−*/*−^ (Plp1-GFP HSCR) mice demonstrate normal appearance of enteric ganglia in the proximal, ganglionated colon (Fig. 3A, 3B) with the expected hypoganglionosis of the transition zone (Fig. 3Bʹ). Distally, there is aganglionosis and prominent hypertrophic nerve bundles that contain GFP positive–cell bodies (Fig. 3Bʹʹ). Immunohistochemical characterization of GFP positive cells within the hypertrophic extrinsic fibers in the aganglionic segment shows an expression of the glial fibrillary acidic protein (GFAP, Fig. 3C, 3Cʹʹʹ) and P75 (Fig. 3E, 3Eʹʹ), but not Tuj1 (Fig. 3D, 3Dʹʹʹ).

The aganglionic segments of 14-day-old HSCR mouse colon were dissected, and the hypertrophic fibers were mechanically separated, dissociated, and sorted for GFP. In culture they formed NLBs expressing GFP (Fig. 3F). Notably, the GFP+ fraction formed significantly more NLBs than the GFP-negative population (Fig. 3G, 4.8 ± 0.6 per 5000 cells vs. 1.0 ± 0.1 per 5000 cells, *n* = 10, ****P < .0001), confirming the proliferation capacity of HSCR-SCs. The GFP+ cells also produced significantly larger NLBs than GFP-negative cells (Fig. 3Gʹ, 83.2 μm ± 4.3 vs. 61.8 μm ± 7.6, *n* = 10, *P < .05). GFP+ NLBs were plated on fibronectin-coated coverslips for 7 days in differentiation conditions and robust cell migration and neuronal differentiation were observed (Fig. 3H, Hʹʹ).

### Neurons Generated from Extrinsic Nerve Fibers of the Aganglionic Region Exhibit Ca^2+^ Activity In Vitro and Ex Vivo

**Figure 4.**
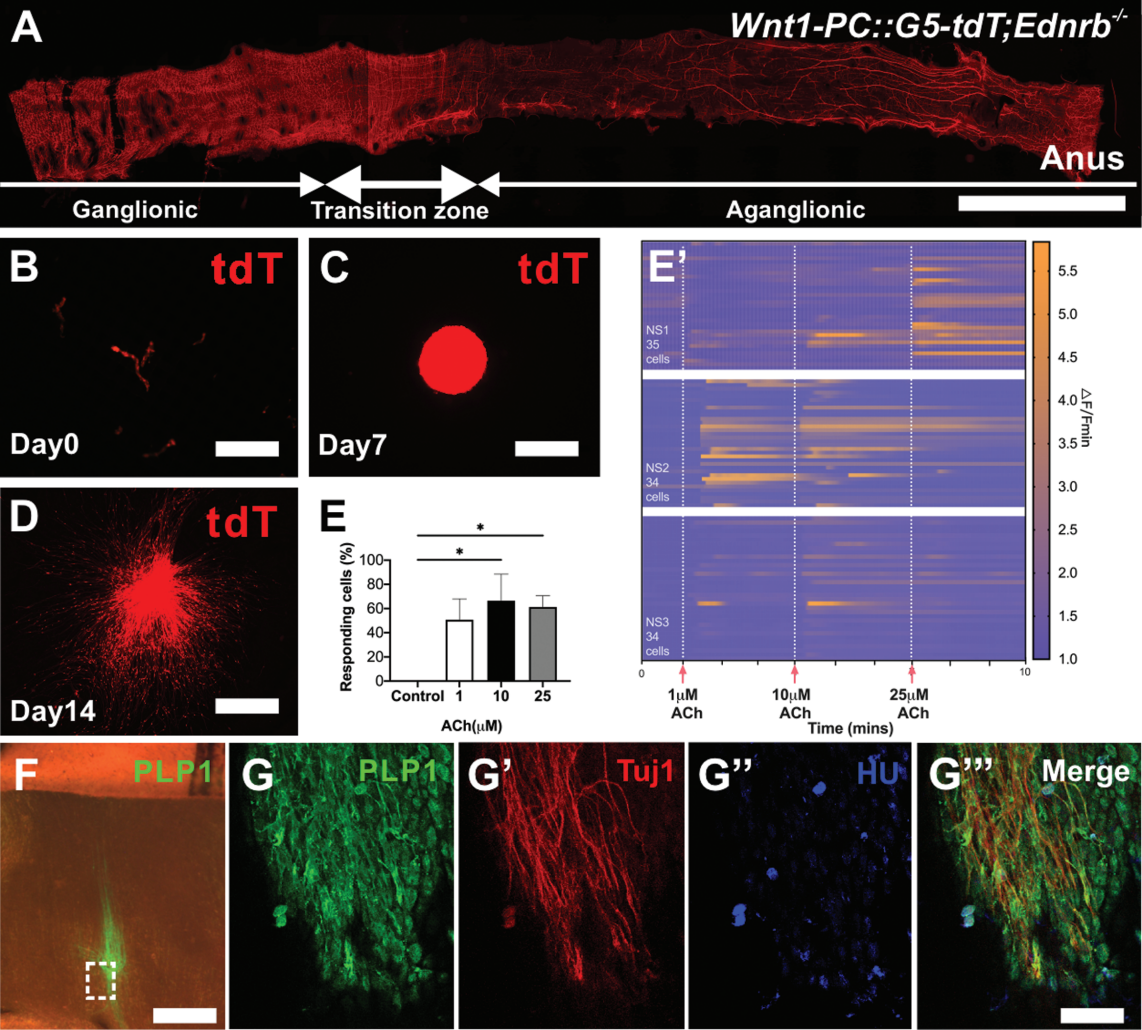
Neurons generated from extrinsic nerve fibers of the aganglionic region exhibit Ca^2+^ activity in vitro and can proliferate and differentiate into neurons following transplantation to aganglionic mouse colon ex vivo. (**A**) Representative image of colon from *Wnt1-PC::G5-tdT;Ednrb*^*−/−*^ mouse. Hypertrophic fibers in the aganglionic segment are separated mechanically (**B**) and cultured to form tdT^+^ NLBs (C). Following 14-day culture on fibronectin, prominent cell migration with extensive fiber projections are seen (**D**). (**E**) Quantification of the percentage of GCaMP5 positive cells that respond under control (unstimulated) conditions, and with 1 μM, 10 μM or 25 μM ACh (*n* = 3, *P < .05). (**E**ʹ) Calcium dynamics of cells with different concentrations of ACh (1, 10, and 25 μM, vertical white dashed lines). Data are represented as mean ± standard deviation from 34 to 35 cells per group with one-way analysis of variance. Changes in GCaMP5 fluorescence are represented on a colorimetric scale. PLP1 positive Schwann cells from hypertrophic nerves in HSCR mice are transplanted to aganglionic colon of HSCR mouse that is grown in culture ex vivo where extensive cell migration and fiber projections are seen (**F**). Immunohistochemistry demonstrates their neuronal differentiation in wholemount preparation (**G**, dotted box, **G-G**ʹʹʹ, arrows). Scale bars, 1cm (A), 100 μm (B-D, F), and 200 μm (G-Gʹʹʹ).

To determine the functionality of HSCR-SCs-derived neurons, we generated a model of HSCR in which neural crest-derived cells express the genetically encoded calcium indicator, GCaMP5 by crossing *Wnt1::Cre;Ednrb*^*+/−*^ mice and *PC::G5-tdT*^*+/−*^*;Ednrb*^*+/−*^ mice (Table 1). Ten to 14 day-old *Wnt1-PC::G5-tdT;Ednrb*^*−/−*^ (Wnt1-G5-tdT HSCR) mice exhibited distal aganglionosis with prominent hypertrophic nerve bundles (Fig. 4A). These tdT positive extrinsic hypertrophic fibers were separated mechanically (Fig. 4B) using the same method as above. Following enzymatic dissociation, these cells were cultured to form NLBs (Fig. 4C). Following culture on fibronectin for 2 weeks, significant cell migration and extensive fiber projections were seen (Fig. 4D). Live cell imaging was performed as previously described.^28^ Changes of GFP intensity, indicative of calcium activity, in up to 35-selected cells in 3 separate preparations were evaluated in response to varying doses of acetylcholine (ACh, Fig. 4Eʹ), demonstrating activation in a significant proportion of cells as compared to control (Fig. 4E, *n* = 3, *P* < .05, one-way ANOVA).

To determine the capacity of HSCR-SCs to migrate and differentiate into neurons within the aganglionic gut environment, these cells were implanted into explanted aganglionic colon obtained from 10-day-old HSCR mice and cultured ex vivo. After 7 days, PLP1-GFP positive HSCR-SCs migrated and projected fibers extensively (Fig. 4F), with immunoreactivity to Tuj1 (Fig. 4Gʹ) and HU in the transplanted cells (Fig. 4Gʹʹ, arrows), confirming neuronal differentiation. We also isolated GCaMP5^+^ HSCR-SCs from 10-day-old Wnt1-G5-tdT HSCR mice and transplanted them into aganglionic colon as above. Successful engraftment and cell migration were seen following 7-day culture ex vivo (Supplementary Fig. S1A) and live cell imaging showed Ca^2+^ activity of transplanted cells in response to electrical field stimulation (Supplementary Fig. S1Aʹ, S1Aʹʹʹ). These observations confirmed that HSCR-SCs possess migratory and neurogenic potential following transplantation to explants of aganglionic smooth muscle ex vivo.

### Transplanted HSCR-SCs Differentiated into Functioning Neurons in Aganglionic Colon In Vivo

**Figure 5.**
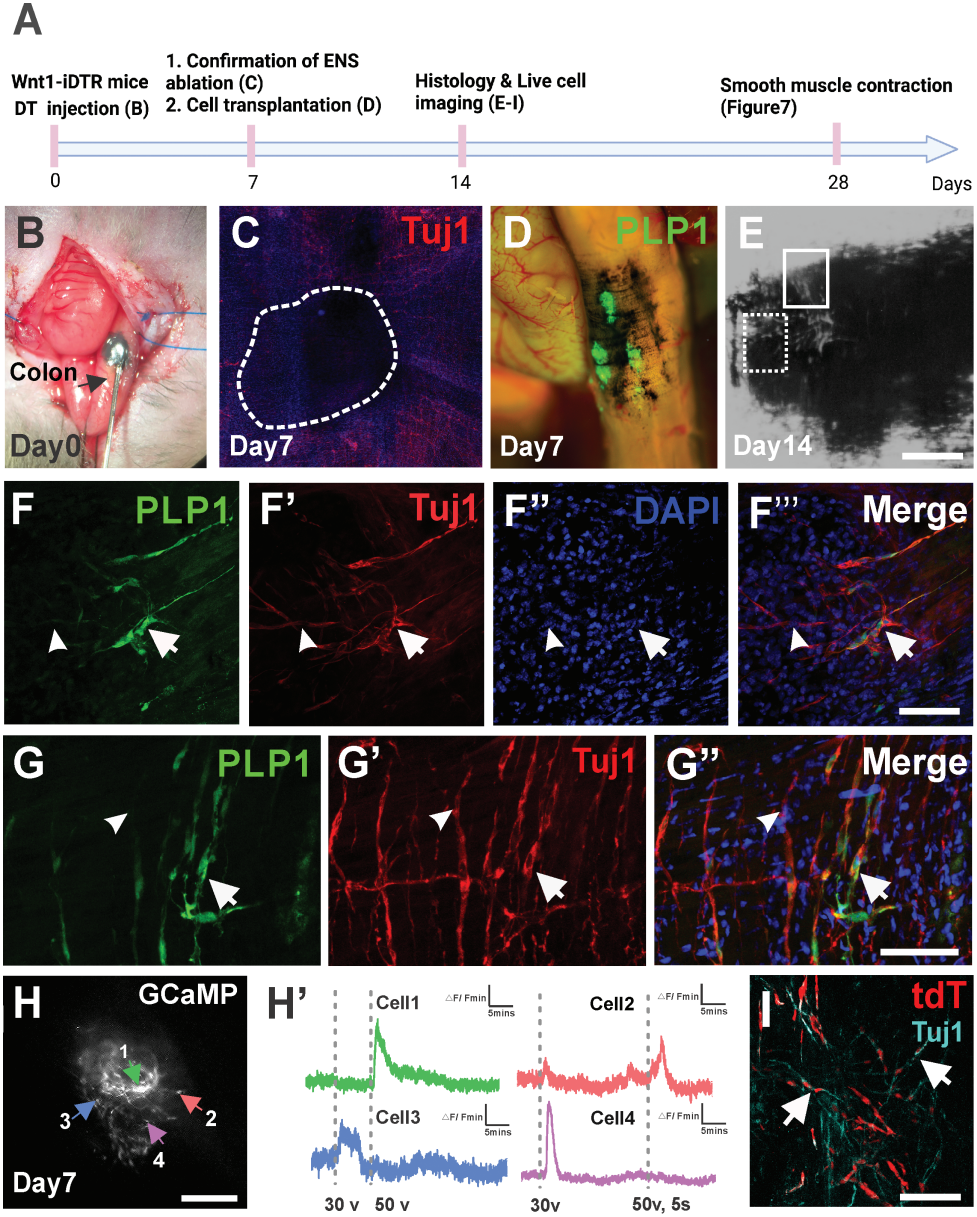
Hypertrophic nerves-derived PLP1-GFP Schwann cells (HSCR-SCs) engrafted and differentiated into functional neurons following transplantation to the aganglionic colon in vivo. (**A**) Experimental overview using Wnt1-iDTR mouse in which all ENS express receptors for diphtheria toxin (DT). (**B**) Focal injection of DT with India ink to mid-colon of Wnt1-iDTR mouse (arrow) on day 0 to create colonic aganglionosis (**C**, dotted line). One week after DT injection (A, day 7), hypertrophic nerves-derived PLP1-GFP Schwann cells (HSCR-SCs) are transplanted to aganglionic region indicated by previous injection of India ink (**D**) and their successful engraftment is confirmed by histological examination 7 days after transplantation (**A**, day 14 and **E**-**Gʹʹ**’’). Subpopulation of transplanted cells (E, dotted line box) give rise to Tuj1^+^ neurons (**F**-**Fʹʹʹ**, arrows) and GFP- Tuj1+ endogenous neuronal fibers are also seen peripheral to the ablated area (**F**-**Gʹʹ**, arrowheads). High power image of cell transplantation site (**E**, line box) shows integration of endogenous and transplanted cell-derived neurons (**G**-**Gʹʹ**, arrows). Live cell imaging on transplanted Wnt1-GCaMP5-tdT cells isolated from hypertrophic nerves is performed 7 days following transplantation (A, day 14 and **H**) demonstrates variable responses to the EFS stimulation (**Hʹ**). Immunohistochemical staining of whole mount recipient colon shows successful ablation of endogenous ENS where transplanted Wnt1-tdT cells are engrafted (**I**, white dotted line) and differentiated into neurons in vivo (**Iʹ** Tuj1, arrows). Scale bars, 100 μm (C, E, H, I), 200 μm (F-G**ʹʹ**, I**ʹ**).

We next sought to investigate whether HSCR-SCs can generate functioning neurons within the aganglionic mouse colon in vivo. We utilized a non-lethal mouse model of colonic aganglionosis created by focal injection of diphtheria toxin (DT) to the mid-colon of 3-month-old Wnt1-iDTR mice^23^ (Fig. 5A, 5B). Seven days following DT injection, successful ablation of the ENS was confirmed by immunostaining for Tuj1 (Fig. 5C, dotted circle). PLP1-GFP positive HSCR-SCs were injected into this aganglionic region 7 days after DT injection (Fig. 5A, 5D). Seven days later (14 days after DT injection), successful engraftment of GFP+ HSCR-SCs was observed, with evidence of neuronal differentiation and fiber extension (Fig. 5F, 5Fʹʹʹ, arrows). Further examination revealed physical proximity between transplanted HSCR-SCs-derived neurons and endogenous Tuj1+ GFP- enteric neuronal fibers (Fig. 5F, 5G, arrowheads, and Supplementary Video 1). Furthermore, 7 days after transplantation of Wnt1-G5-tdT positive HSCR-SCs, the recipient colon was dissected and the wholemount smooth muscle layer was prepared for live cell imaging of transplanted HSCR-SCs. Ca^2+^ activity of tdT+ HSCR-SCs was observed in response to EFS (Fig. 5H, 5Hʹ, Supplementary Video 2). Cells exhibiting a fast upstroke in calcium influx immediately upon EFS followed by a biexponential decay were observed, consistent with the properties of enteric neurons^29^ (Fig. 5Hʹ, Cells 1 and 4). Finally, immunohistochemical staining of whole-mount preparations of the recipient aganglionic colon suggested successful engraftment of tdT–positive transplanted cells and their neuronal differentiation in vivo (Fig. 5I, arrows).

### Transplantation of HSCR-SCs Restores Contractility of Aganglionic Smooth Muscle

**Figure 6.**
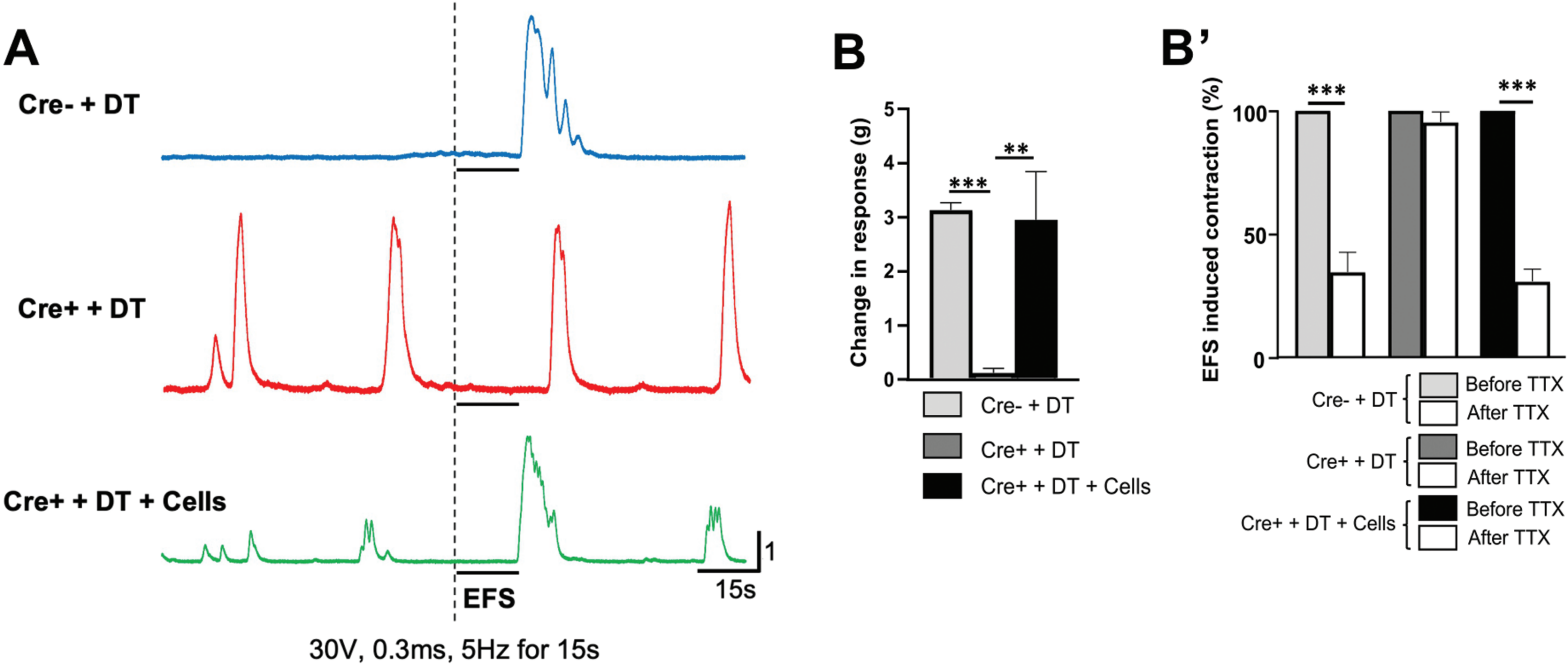
Improvement of muscle contraction in aganglionic colon by transplantation of HSCR-SCs. (**A**) Representative traces of colonic contractile force in Wnt1::Cre^-^;R26-iDTR that receives DT injection (A, Cre- + DT, blue line), Wnt1::Cre^+^;R26-iDTR (Cre+) with DT injection (A, Cre+ + DT, red line) and Cre+ + DT followed by HSCR-SCs transplantation (A, Cre+ + DT + Cells, green line). Voltage-dependent changes in contractile force of colonic muscle preparation after EFS (30 V, 0.3 ms 5H z for 30 s) showed cell transplantation significantly improved muscle contractile activity (**B**, *n* = 4, ***P < .001, **P < .01) This response was significantly reduced by the presence TTX. (**Bʹ**, *n* = 4, * *P* < .05). Data are represented as mean ± SD from 4 mice per group by Student’s *t*-test. Abbreviations: TTX, tetrodotoxin; EFS, electrical field stimulation.

Recipient mice were maintained for 21 days following cell transplantation to examine the contractility of aganglionic smooth muscle using an organ bath system (Fig. 5A, 4 weeks after DT injection). Basal contractile patterns were recorded from three groups of mice: Cre negative mice that received DT (Fig. 6A, Cre- + DT “control” group, *n* = 4), Wnt1-iDTR Cre+ mice with DT (Cre+ + DT “aganglionosis” group, *n* = 4), and Wnt1-iDTR Cre+ mice with DT followed by HSCR-SCs transplantation (Cre+ + DT + Cells “aganglionosis + cells” group, *n* = 4). EFS (30 V, 0.3 ms, 5 Hz for 15 s, arrow) was applied while recording.

In the control group, a rebound contraction in response to EFS was seen, followed by relaxation/quiescence (Fig. 6A, Cre- + DT). In contrast, aganglionic smooth muscle showed no response to the EFS, whereas rhythmic, spontaneous, myogenic contractions were observed (Fig. 6A, Cre+ + DT). Smooth muscle from the “aganglionosis + cells” colon exhibited restoration of EFS-induced contractile activity (Fig. 6A, Cre+ + DT + Cells), similar to “control” colon. Quantitative analysis showed that colonic tissue from aganglionic mice displayed a marked reduction in contractile activity (Fig. 6B, 3.1 ± 0.07 g in control vs 0.10 ± 0.04 g in aganglionosis; ***P < .001, *n* = 4), which was restored by cell transplantation (Fig. 6B, 2.9 ± 0.5 g in Cre+ + DT + cells; **P < .01, *n* = 4).

Muscle contraction was also recorded in the presence of tetrodotoxin (TTX, 0.5 μM, Alomone Labs, Israel), a voltage-gated sodium channel blocker. No changes were seen in muscle contraction in the aganglionic colon before and after addition of TTX (Fig. 6Bʹ, Cre+ + DT). However, the contractile response to EFS was significantly reduced by addition of TTX in the Cre- + DT control colon (Fig. 6Bʹ, 34.5 ± 4.7%; ***P < .001) and “aganglionosis + cells” group (Fig. 6Bʹ, 30.7 ± 2.9%; ***P <.001), suggesting that restoration of muscle contraction in HSCR-SC transplanted aganglionic colon is mediated by the transplanted neurons.

### Isolation and Characterization of Human HSCR-SCs and Their Transplantation to Aganglionic Mouse Colon *In Vivo*

**Figure 7.**
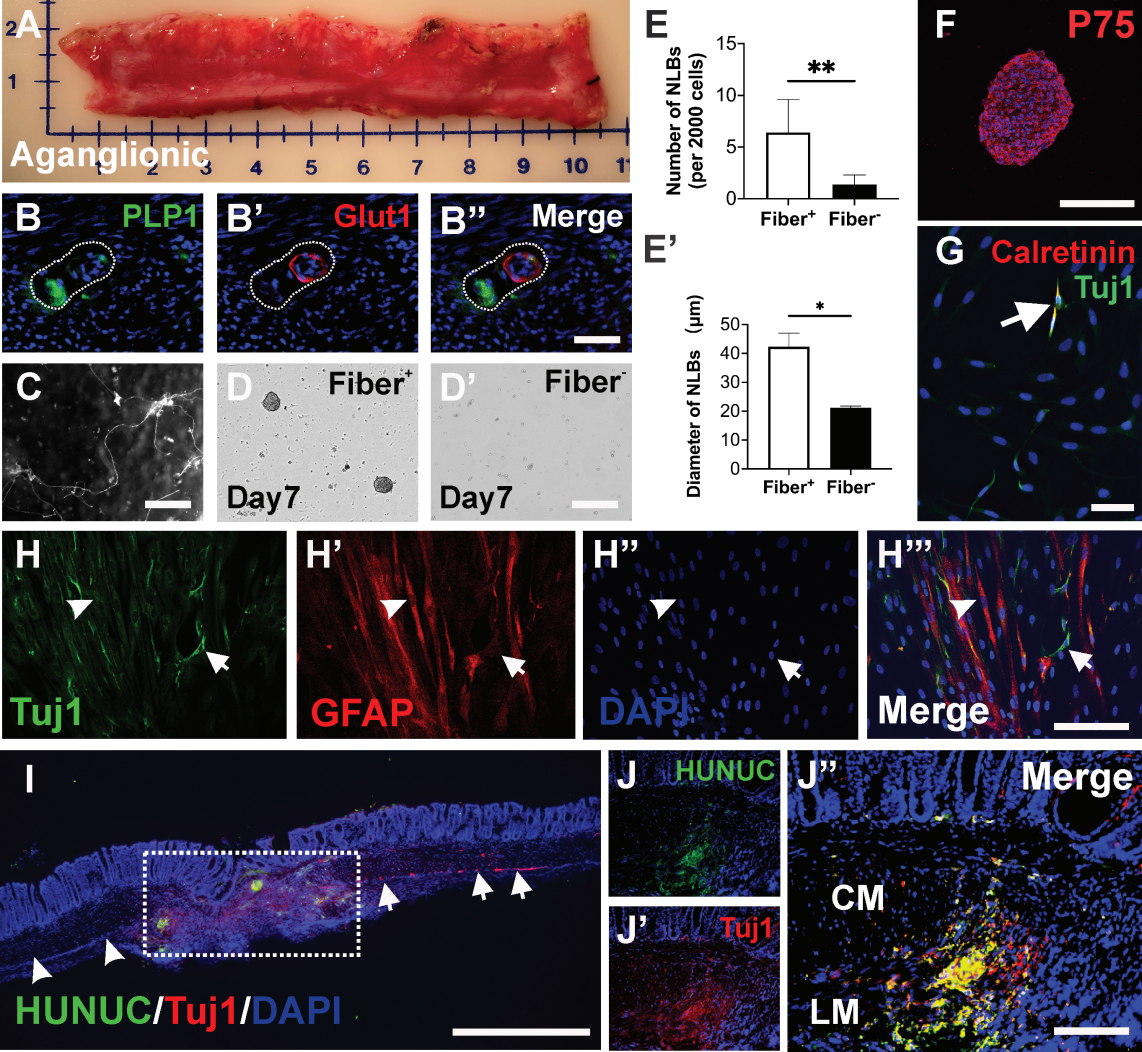
Isolation and characterization of human HSCR-SCs and their transplantation to colonic aganglionosis in vivo. (**A**) Aganglionic colonic segment surgically resected from 6-month-old boy with HSCR. Glut1 positive extrinsic-derived nerve fibers contain PLP1-expressing cells. (**B**-**Bʹʹ**). Extrinsic fibers are separated (**C**), dissociated and plated for further culture. The cells from these extrinsic fibers generate more (**D**, **E**, *n* = 4, **P < .01) and larger (**Dʹ**, **Eʹ**, *n* = 4, *P < .05) NLBs than non-extrinsic fiber-derived cells. Immunohistochemical characterization of human NLBs demonstrates these NLBs contain P75^+^ neural crest-derived cells (**F**). Culturing these NLBs on fibronectin showed their capacity to differentiate into Tuj1^+^ neurons (**G** and **H**, arrows) and glial cells (**H**-**Hʹʹʹ**, arrowheads) in vitro. Subpopulation of differentiated neurons is also immunoreactive for calretinin (G, arrows), an enteric neuron subtype. Seven days following transplantation of these human NLBs to aganglionic mouse colon in vivo, staining for nuclei marker (**I**, HUNUC) identified transplanted human HSCR-SCs within the aganglionic region (I, dotted box). Immunohistochemical staining shows successful ablation of human enteric neurons (I, arrowheads) and endogenous myenteric neurons in non-ablated area of recipient colon (I, arrows) and neuronal differentiation of transplanted human HSCR-SCs (**Jʹ**-**Jʹʹ**, Tuj1, red) in vivo. Data are represented as mean ± SD per group by Student’s *t*-test. Scale bars 1 mm (I), 100 μm (B-B**ʹʹ**, D-D**ʹ**, G, H-H**ʹʹ**, J-J**ʹʹ**), 200 μm (C, F). Abbreviations: CM, circular muscle; LM, longitudinal muscle.

To test the feasibility of isolating and culturing human HSCR-SCs, 4 patients with HSCR were enrolled (3 males and 1 female, average age 10 ± 14 months, including 2 children with total colonic aganglionosis). Aganglionic colonic tissues were obtained following surgical resection (Fig. 7A) and with institutional ethical approval. Immunohistochemical examination of human aganglionic colon showed glucose transporter 1 (GLUT1) positive extrinsic-derived nerve fibers (Fig. 7B), with adjacent PLP1 positive Schwann cells (Fig. 7B, 7Bʹʹ). These extrinsic fibers were collected and enzymatically dissociated as above (Fig. 7C). Following 10-14 days in culture, NLBs were formed in the fiber-derived cell cultures (Fig. 7D, Dʹ), where the fiber negative fraction formed significantly fewer (Fig. 7E, 6.4 ± 1.4 per 2000 cells vs. 1.4 ± 0.4 per 2000 cells, **P <.01, *n* = 4) and smaller (Fig. 7Eʹ, 42.3 ± 4.8 μm vs. 21.2 ± 0.5 μm, *P <.05, *n* = 4) NLBs, confirming marked enrichment of proliferative cells from the extrinsic nerves in human aganglionic intestine. Immunohistochemical characterization demonstrated that these NLBs contain P75^+^ neural crest-derived cells (Fig. 7F) capable of differentiation into neurons (Tuj1+, arrows in Fig. 7G, 7H) and glia (GFAP+, Fig. 7H, 7Hʹʹʹ, arrowheads) in culture. A subpopulation of differentiated neurons was also immunoreactive for calretinin (Fig. 7G, arrow). Finally, these human HSCR-SCs were transplanted to aganglionic mouse colon in vivo. Colonic aganglionosis was generated by focal injection of DT to the mid-colon of 3-month-old Wnt1-iDTR mice as described above (Fig. 5A, 5C). Seven days following DT injection, human HSCR-SCs were microinjected into the region where the ENS has been ablated. Recipient colon was processed for immunohistochemistry 7 days after cell transplantation. Human-derived cells, identified by immunoreactivity to a human nuclear marker (Fig. 7I, HUNUC, dotted box), engrafted and migrated within the aganglionic segment (Fig. 7I, arrowheads). Tuj1 staining demonstrated endogenous myenteric neurons in the non-ablated area (Fig. 7I, arrows) and suggested neuronal differentiation of the transplanted cells (Fig. 7J, 7Jʹʹ). Transplanted cells were also immunoreactive for the presynaptic protein, Synapsin 1 (Supplementary Fig. S2).

## Discussion

In this study, we focus on PLP1-expressing Schwann cells (SCs) residing along the hypertrophic nerve fibers in the aganglionic colon of mice and humans with HSCR. Using cell culture, live cell imaging, ex vivo and in vivo transplants, and organ bath experiments, we show that HSCR-SCs can be isolated and expanded in culture, where they demonstrate neurogenic potential. Moreover, these cells are able to engraft within the aganglionic gut environment following in vivo transplantation, give rise to functioning neurons, and restore contractile activity in recipient smooth muscle. These observations are highly supportive of the potential for utilizing the aganglionic bowel as an autologous source for cell-based treatment of HSCR.

In HSCR, the aganglionic segment is devoid of intrinsic enteric ganglia, but extrinsic innervation persists. This extrinsic innervation arises from autonomic ganglia in the pelvis, spinal sensory ganglia, and paravertebral sympathetic ganglia.^30^ Sacral extrinsic fibers projecting from the pelvic ganglia ascend in the perirectal connective tissue and between the muscle layers of the colon where they intersect with vagal neural crest-derived cells.^31^ At the most distal part of the aganglionic region, extrinsic fibers and cells from pelvic ganglia radially penetrate the muscularis propria to reach the submucosa and mucosa.^32^ Hypertrophy of extrinsic nerves is a well-described feature in patients with HSCR, and appearance of hypertrophic nerve trunks in rectal suction biopsy specimens is one of the diagnostic criteria of HSCR.^33^ Although the pathophysiological mechanisms leading to extrinsic nerve hypertrophy are uncertain, histochemical studies of aganglionic bowel show that these large nerve trunks are immunoreactive for acetylcholinesterase^34^ and GLUT1.^27,35^ Several previous reports utilizing mouse models of HSCR have demonstrated extrinsic fibers in the aganglionic colon comprising neural crest-derived nerve fibers^31,36^ and Schwann cells.^16,21^ Wilkinson et al. have shown that P75+ neural crest-derived cells can be isolated from an aganglionic segments of human HSCR and expanded in culture.^13^ We also observed tdTomato expression in the hypertrophic nerve bundles within the aganglionic segment of Wnt1-G5-tdT HSCR mice, confirming that these fibers contain neural crest-derived cell bodies. These and our data suggest that a pool of undifferentiated precursors with neurogenic potential is present in the aganglionic bowel.

There is growing interest in understanding the mechanism of postnatal enteric neurogenesis. PLP1 is the major myelin protein and widely expressed in enteric glial cells,^26^ SCs, and Schwann cell precursors (SCPs).^37^ SCPs residing in the extrinsic intestinal nerves have been shown to be an additional source of enteric neurons pre- and postnatally.^15,21^ In the current study, we demonstrate the presence of PLP1-positive SCs within the hypertrophic nerve bundles along the aganglionic colon and show their successful expansion in culture. Interestingly, the formation of NLBs from the PLP1-negative population is also seen, which may be caused by neuronal progenitors that do not express PLP1. Previous studies have reported 75%-99.5% overlap^26,38^ between PLP1 and other glial/SC markers, including GFAP, S100, or Sox10. We also confirmed their neuronal differentiation property in culture and the following transplantation to the aganglionic gut environment. In our immunohistochemical analysis, we often see colocalization of GFP and neuronal markers, such as Tuj1 and PGP9.5. It has been reported that GFP expression can persist for some time since its half-life is about 26 h.^39^ Therefore, even after a cell undergoes neuronal differentiation and downregulates PLP1 expression, the cytosolic GFP protein can persist.

Recently, Uesaka et al. demonstrated that 20% of enteric neurons in the mouse colon are derived from SCPs utilizing *desert hedgehog* (*Dhh*)*::Cre*-mediated genetic labeling. *Dhh* is expressed in SCPs that invade the gut and generate enteric neurons even in the absence of Ret signaling.^15^ It was also shown that the SCs residing in the extrinsic nerves in mouse models of HSCR can be activated by a reduction in the intrinsic neurons^21^ or rectal administration of GDNF.^16^ Soret et al.^16^ gave rectal GDNF enemas to early postnatal HSCR mice and found induction of neurogenesis from Sox10+ SCs residing in the extrinsic fibers, reversing the aganglionic phenotype and improving colonic motility and survival. These remarkable observations were reproduced in ex vivo organ cultures using aganglionic gut explants from human HSCR patients.^16^ These important studies support our observations that transplanted SCs can give rise to functioning neurons within the aganglionic colon. We utilized cell transplantation techniques combined with live cell imaging and organ bath electrophysiology to validate the functional contribution of transplanted Schwann cells to smooth muscle contractility. This is the first study to demonstrate improvement in aganglionic smooth muscle contraction using cells isolated from the aganglionic region, positioning an alternative to the in situ strategy of Soret et al.^16^ Our cell-based approach does not require drug administration to the child and it allows donor cells to be isolated, expanded, and frozen for future use, including treatment of post–pullthrough problems or performing drug screening in vitro.

Interestingly, in our study, human HSCR-SCs engrafted and survived remarkably well after transplantation to the aganglionic colon of Wnt1-iDTR mice induced by DT in vivo, although we encountered difficulties in quantifying cell engraftment and the efficiency of forming functioning neurons following transplantation. These challenges are due to a couple of factors: i) the number of transplanted cells is variable since we are transplanting neurospheres, which each contain variable numbers of cells and ii) quantifying the number of engrafted cells is difficult since many remain highly concentrated within the transplanted neurosphere and cannot be quantified accurately. Interestingly, we have found no correlation between cell engraftment or cell coverage and functional recovery as measured by EFS or GI motility in our unpublished observations from multiple prior studies.

Several mouse models of colonic aganglionosis exist,^40,41^ but most of these transgenic animals are lethal before or soon after birth, preventing long-term assessment of the effects of transplanted cells on gut motility. We therefore used a DT-mediated, non-lethal model of focal aganglionosis, as we previously described.^23^ This model is useful for evaluating neuronal cell function and bowel contractility but cannot demonstrate improvement in colonic motility in vivo since the focal ENS deficit induced by DT does not result in measurable dysmotility in vivo. While Fattahi et al. observed improved survival following transplantation of pluripotent stem cell-derived enteric neural progenitors to HSCR mice,^42^ improved colonic functions was not demonstrated. To date, there is no published evidence that cell transplantation can restore the motility of aganglionic colon in vivo, although our muscle contractility results are promising. This is a gap that needs to be filled prior to the clinical application of this technology.

In summary, this study demonstrates the significant translational potential of PLP1+ Schwann cells residing along the extrinsic fibers in the aganglionic colon. Isolating them from the aganglionic bowel normally removed during surgery for HSCR will provide an autologous source of cells that could be transplanted back to the patient in those cases complicated by colonic dysmotility due to residual aganglionosis or a transition zone pullthrough.

## Supplementary Material

szac076_suppl_Supplementary_Figure_S1

szac076_suppl_Supplementary_Figure_S2

szac076_suppl_Supplementary_Table_S1

szac076_suppl_Supplementary_Video_S1

szac076_suppl_Supplementary_Video_S2

## Data Availability

The data that support the findings of this study are available from the corresponding author upon reasonable request.
