# Supplementary figures and images for: Schwann Cells in the Aganglionic Colon of Hirschsprung Disease Can Generate Neurons for Regenerative Therapy
Source: Stem Cells Transl Med. 2022 Nov 2;11(12):1232–44. doi: 10.1093/stcltm/szac076 (PMC9801298; doi:10.1093/stcltm/szac076)

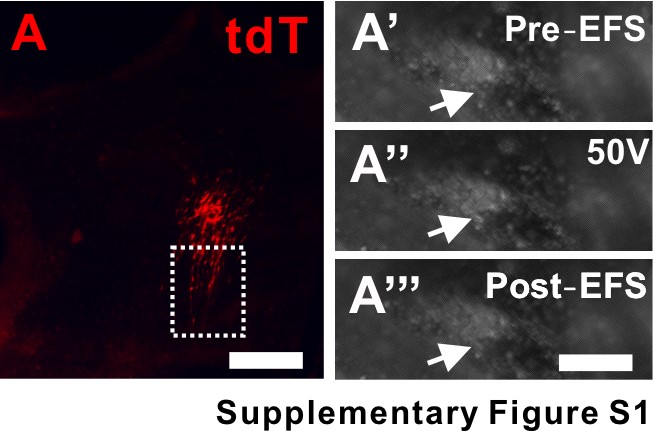

Supplement: szac076_suppl_Supplementary_Figure_S1 [file szac076_suppl_supplementary_figure_s1.jpeg]

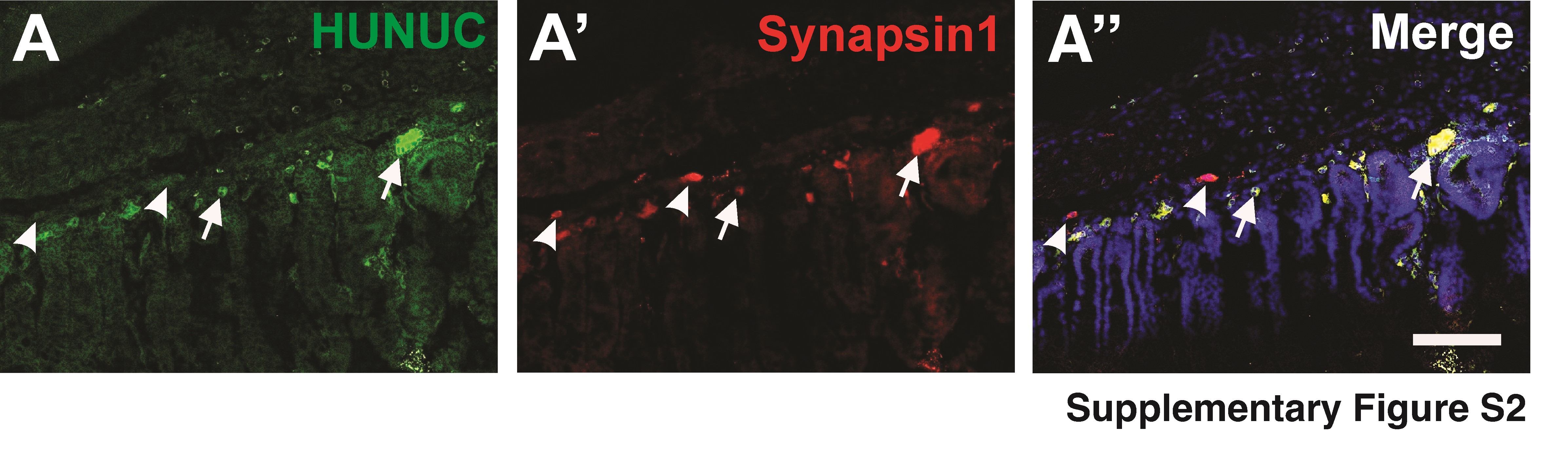

Supplement: szac076_suppl_Supplementary_Figure_S2 [file szac076_suppl_supplementary_figure_s2.jpeg]
