# Supplementary material for: Schwann Cells in the Aganglionic Colon of Hirschsprung Disease Can Generate Neurons for Regenerative Therapy
Source: Stem Cells Transl Med. 2022 Nov 2;11(12):1232–44. doi: 10.1093/stcltm/szac076 (PMC9801298; doi:10.1093/stcltm/szac076)
Supplement: szac076_suppl_Supplementary_Table_S1 [file szac076_suppl_supplementary_table_s1.docx]

Supplementary table 1.

| Mouse nomenclature | Common Name | Source | References |
| --- | --- | --- | --- |
| B6.129S4(Cg)-*Mapt^tm1(EGFP)Klt^*/J | *Tau^GFP^* | JAX 029219 | Tucker 2001 (PMID 11135642) |
| B6;CBA-Tg(Plp1-EGFP)10Wmac/J | *Plp1-GFP* | Wendy Macklin University of Colorado, Denver | Mallon et al. 2002 (PMID: 11826117) |
| B6;129-*Ednrb^tm1Ywa^*/J | *Ednrb^+/-^* | JAX 003295 | Hosoda et al. 1994 (PMID: 8001159) |
| B6.Cg-*H2az2^Tg(Wnt1-cre)11Rth^* Tg(Wnt1-GAL4)11Rth/J | *Wnt1-Cre* | JAX 009107 | Danielian et al. 1996 (PMID: **8848044)** |
| B6; 129S6- *Polr2a^tnm1(CAG-GCaMP5g;-tdTomato)Tvrd^*/J | *PC::G5-tdTtomato* (*PC::G5-tdT*) | JAX 024477 | Gee et al 2014 (PMID: 25155958) |
| C57BL/6-*Gt(ROSA)26Sor^tm1(HBEGF)Awai^*/J | *R26-iDTR* | JAX 007900 | Buch et al. 2005 (PMID: 15908920) |
